# Intracranial epidural hematoma follow-up using bidimensional ultrasound

**DOI:** 10.5935/0103-507X.20170036

**Published:** 2017

**Authors:** Fabio Holanda Lacerda, Hassan Rahhal, Leonardo Jorge Soares, Francisco del Rosario Matos Ureña, Marcelo Park

**Affiliations:** 1 Unidade de Terapia Intensiva, Departamento de Emergências Clínicas, Hospital das Clínicas, Faculdade de Medicina, Universidade de São Paulo - São Paulo (SP), Brasil.

Bidimensional encephalic ultrasound can be used to diagnose several types of lesions as epidural hematomas.^(1)^ To illustrate this use, we present a patient in which an epidural hematoma was monitored through the use of a hemicraniectomy bidimensional ultrasound.

A 28-year-old male patient was found unconscious after a fall from a platform. He was promptly given medical attention on-site by the local pre-hospital emergency system. When evaluated by the emergency medical team, his Glasgow Coma scale was 3. The medics performed a tracheal intubation, placed an intravenous catheter, and immobilized the patient's neck and body before transport to the emergency room. Upon admission, the patient had a secure airway, stable hemodynamics, and normal assisted ventilation; his right ear was bleeding, and he had miotic pupils.

**Figure 1:**
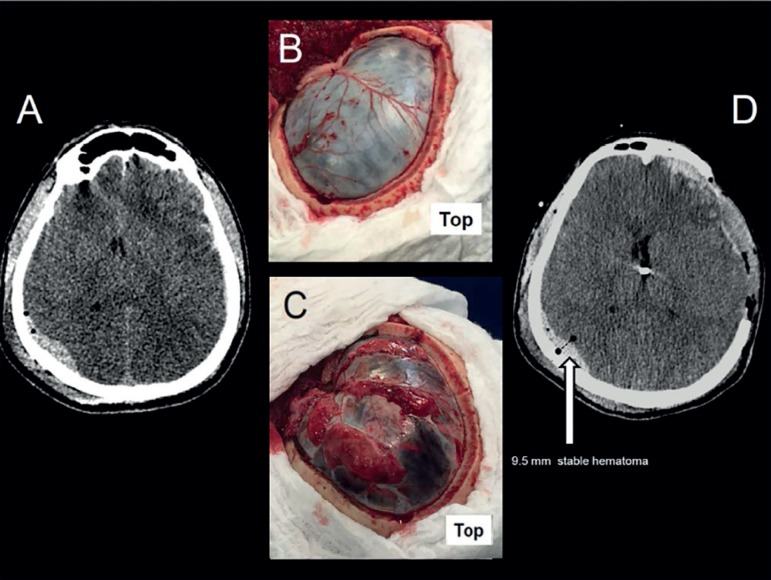
Cranial computerized tomography scans and surgical aspects. (A) Initial computerized tomography scan showing diffuse edema with left frontotemporal subdural hematoma and right occipital epidural hematoma. (B) Post-craniotomy aspect. (C) Post-duraplasty aspect, depicting severe cerebral edema. (D) Computerized tomography scan after surgery with a 9.5mm diameter epidural hemorrhage.

The initial computed tomography (CT) showed diffuse cerebral edema, with a left frontotemporal subdural hematoma, and a right occipital epidural hematoma (Figure 1A). The neurosurgical team planned to install an intracranial (intraventricular) pressure (ICP) monitor, drain the subdural hematoma, and perform a decompressive hemicraniectomy (Figure 1B). During the surgery, the patient developed a profound hemodynamic instability requiring high dosages of norepinephrine and vasopressin, and preventing the surgical team from draining the epidural hematoma. Immediately after the surgical procedures, ICP reduced from 46.8mmHg to 12mmHg. A CT showed an improvement of central line lateral displacement, and the stability of the epidural hematoma (Figure 1). After 24 hours in the intensive care unit (ICU), the ICP remained < 20 mmHg, and a repeat CT showed that the basal cisterns were clearly open. Sedation was suspended to allow a neurological examination. The patient awoke with a severe aphasia but was able to control his airway, allowing extubation 36 hours after ICU admission.

**Figure 2:**
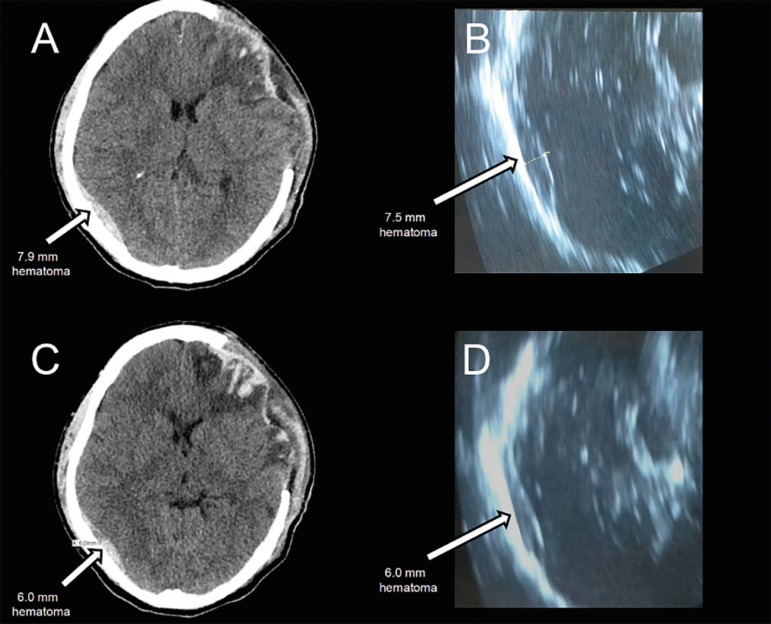
Cranial computerized tomography ultrasound scans following surgery. (A) Day 2 computerized tomography scan showing persistent diffuse edema and epidural hemorrhage, approximately 7.9mm diameter. (B) Day 2 ultrasound scan showing an epidural hematoma with 7.5mm diameter. (C) Day 3 computerized tomography scan showing an epidural hematoma with 6.0mm diameter. (D) Day 3 ultrasound scan showing a 6.0mm diameter hematoma.

The epidural hematoma was monitored through a bedside CT trans-hemicraniectomy ultrasonography unit (LOGIQ P5 system, General Electric Healthcare, with a curvilinear transducer of 5MHz) for two days (day 2 - Figures 2A and 2B, and day 3 - Figures 2C and 2D). Afterwards, as the ultrasound and tomography measurements appeared to agree, only ultrasonography was used for further monitoring. The patient was discharged uneventfully from the ICU 7 days after admission.

Ultrasound enables the diagnosis of an epidural hematoma with^(1)^ or without prior hemicraniectomy.^(2)^ Furthermore, measurement of an epidural hematoma appears to be reliable using ultrasound in hemicraniectomized patients.^(1)^ There is no literature supporting the safety of temporal evaluation of intracranial hematomas using ultrasound. However, it seems feasible as demonstrated by our experience, and could potentially be safer and cost efficient by reducing unnecessary CT scans in severe brain trauma patients.^(3)^
